# Strategic Positioning of Connexin36 Gap Junctions Across Human Retinal Ganglion Cell Dendritic Arbors

**DOI:** 10.3389/fncel.2018.00409

**Published:** 2018-11-22

**Authors:** Orsolya Kántor, Gergely Szarka, Zsigmond Benkő, Zoltán Somogyvári, Emese Pálfi, Gábor Baksa, Gergely Rácz, Roland Nitschke, Gábor Debertin, Béla Völgyi

**Affiliations:** ^1^Department of Neuroanatomy, Faculty of Medicine, Institute for Anatomy and Cell Biology, Albert-Ludwigs-University Freiburg, Freiburg, Germany; ^2^MTA-PTE NAP 2 Retinal Electrical Synapses Research Group, Pécs, Hungary; ^3^Department of Anatomy, Histology and Embryology, Semmelweis University, Budapest, Hungary; ^4^Department of Experimental Zoology and Neurobiology, University of Pécs, Pécs, Hungary; ^5^Center for Neuroscience, University of Pécs, Pécs, Hungary; ^6^János Szentágothai Research Center, University of Pécs, Pécs, Hungary; ^7^Complex Systems and Computational Neuroscience Group, Wigner Research Center for Physics, Hungarian Academy of Sciences, Budapest, Hungary; ^8^Department of Pathology and Experimental Cancer Research, Semmelweis University, Budapest, Hungary; ^9^Life Imaging Center, Center for Biological Systems Analysis, Albert-Ludwigs University, Freiburg, Germany; ^10^BIOSS Center for Biological Signaling Studies, Albert-Ludwigs-University Freiburg, Freiburg, Germany

**Keywords:** gap junction, electrical synapse, ganglion cell, inner plexiform layer, ganglion cell layer, human retina

## Abstract

Connexin36 (Cx36) subunits form gap junctions (GJ) between neurons throughout the central nervous system. Such GJs of the mammalian retina serve the transmission, averaging and correlation of signals prior to conveying visual information to the brain. Retinal GJs have been exhaustively studied in various animal species, however, there is still a perplexing paucity of information regarding the presence and function of human retinal GJs. Particularly little is known about GJ formation of human retinal ganglion cells (hRGCs) due to the limited number of suitable experimental approaches. Compared to the neuronal coupling studies in animal models, where GJ permeable tracer injection is the gold standard method, the post-mortem nature of scarcely available human retinal samples leaves immunohistochemistry as a sole approach to obtain information on hRGC GJs. In this study Lucifer Yellow (LY) dye injections and Cx36 immunohistochemistry were performed in fixed short-post-mortem samples to stain hRGCs with complete dendritic arbors and locate dendritic Cx36 GJs. Subsequent neuronal reconstructions and morphometric analyses revealed that Cx36 plaques had a clear tendency to form clusters and particularly favored terminal dendritic segments.

## Introduction

Various connexin (Cx) gene sequences emerged throughout vertebrate evolution and their products comprise a populous protein family with over 20 members (Güldenagel et al., 2000; Cruciani and Mikalsen, 2006; Söhl and Willecke, 2003). Cx subunits are widely expressed in both non-neuronal and neuronal tissues, where they form gap junctions (GJ) between connecting cells. GJs connect neurons throughout the central nervous system in all examined mammals, where they allow the passage of signals between neurons (Furshpan and Potter, 1957; Watanabe, 1958). The mammalian retina has been a popular model of GJ studies as a number of Cx subunits are expressed by various retinal neurons, including Cx30.2, Cx36, Cx45, Cx50, and Cx57 (Güldenagel et al., 2001; Lee et al., 2003; Massey et al., 2003; Hombach et al., 2004; Maxeiner et al., 2005; Müller et al., 2010; Han and Massey, 2005). It has been shown, that similar to other mammalian species the human retina expresses Cx36 and Cx45 both in the IPL and OPL (Söhl et al., 2010) and the expression patterns resemble those of other mammalian species, including the mouse, rabbit, and rat (Feigenspan et al., 2001; Mills et al., 2001; Deans et al., 2002; Kihara et al., 2006, 2010; O'Brien et al., 2012; Kovács-Öller et al., 2014, 2017; Kántor et al., 2016a, 2017). Cx36 comprising retinal GJs have been shown to play a number of roles in signal processing, including the transmission-, averaging- and synchronization of signals (Mastronarde, 1983; Brivanlou et al., 1998; DeVries, 1999; Bloomfield and Völgyi, 2009; Völgyi et al., 2013a,b; Roy et al., 2017). Evidence for retinal ganglion cell (RGC) GJs have been found in most RGC types of the examined animal species, (Hidaka et al., 2004; Schubert et al., 2005a,b; Völgyi et al., 2005, 2009, 2013a,b; Pan et al., 2010) indicating that GJs are integrated into most RGC microcircuits and thus they play essential roles in RGC signaling. However, it is unknown whether hRGCs possess similar electrical synaptic connections or they fundamentally differ in this respect from those of their non-human counterparts.

In this study we combined the intracellular injection of Lucifer yellow (LY), Cx36 immunohistochemistry and 3D reconstruction based morphometric analysis to study the Cx36 plaque distribution across the dendritic branches of hRGCs. We found that Cx36 plaque numbers, densities and distribution over dendritic arbors varied considerably in our heterogeneous sample. Despite this heterogeneity of the examined neurons, we also encountered population wide general features including the relative higher density of plaques in terminal dendrite segments and a tendency for plaques to occur in pairs (or triplets). Possible functional aspects regarding these two general phenomena are discussed in this work as well.

## Materials and methods

### Histological preparation

Human donor tissue from patients without reported history of eye disease was collected in accordance with the tenets of Declaration of Helsinki or from cadavers from the 1st Department of Pathology and Cancer Research and from Department of Anatomy, Histology and Embryology, Semmelweis University, Budapest, Hungary. Retinas from 3 patients were investigated in the present study (2 females, 1 male, age: 59–85 years, 3–7 h post-mortem time). All personal identifiers were removed and samples were coded before histological processing. All experimental protocols were approved by the local ethics committee (TUKEB58/2014). To comply with institutional ethics regulations all necessary written consents to utilize human tissue for scientific purposes of this study were obtained from either the donors, or their eligible relatives. After the removal of corneas, posterior eyecups were fixed in 4% buffered paraformaldehyde (PFA) for 2 h at +4°C. Samples were cut into six radial pieces then rinsed several times in 0.1 M phosphate buffered saline (PBS, pH 7.4). Isolated retinal whole mounts (pigment epithelium was carefully removed) were cut in small pieces.

### Intracellular lucifer yellow injections

Borosilicate glass pipettes were filled with 4% Lucifer Yellow die (LY, Sigma-Aldrich, Budapest, Hungary, resistance: 150–500 MΩ). Human RGCs from mid-peripheral (3–6 mm centrality) to peripheral (6–9 mm centrality) locations were injected with LY using a Zeiss Axioscope microscope, 40x water immersion lens, Zeiss micromanipulator (Carl Zeiss Inc., Jena, Germany) and Digitimer iontophoretic dye marker (Digitimer Ltd., Welwyn Garden City, UK; current: −4.5 nA, duration: 10–15 min). Afterwards, retinal pieces were postfixed in 4% PFA with 0.5% glutaraldehyde (Sigma-Aldrich, Budapest, Hungary) for 45 min and rinsed extensively in 0.1 M phosphate buffered saline (PBS, pH 7,4). After the LY injection, each further step was carried out under protection from light. To enhance penetration in whole mounts, tissue was soaked in 30% sucrose and freeze-thawed three times. To remove glutaraldehyde from the tissue, retinas were treated with 1% sodium-borohydride for 30 min (Sigma-Aldrich, Budapest, Hungary).

### Fluorescent immunohistochemistry

Fluorescent Cx36 immunohistochemical reactions on the specimens with LY filled neurons were carried out according to standard protocols (Kántor et al., 2016a). Briefly, tissues were washed several times with PBS (25 mM with 0.2% Triton-X, PBS-TX). Non-specific background staining was blocked in 10% donkey serum diluted in PBS-TX. Specimens were then incubated in the anti-Cx36 primary antibody (mouse anti-connexin 35/36 1:1,000; Merck Ltd, cat# MAB3045, clone 8F6.2) at +4°C (72 h). After extensive rinsing, specimens were incubated overnight at 4°C with the anti-mouse IgG secondary antibody conjugated with Alexa 647 (1:500, Life Technologies, Hungary) diluted in PBS-TX and 3% normal donkey serum. After several rinsing steps, whole mounts were mounted on gelatin coated slides and all specimens were coverslipped using AquaPolymount (Polysciences Europe GmbH, Eppelheim, Germany) as mounting medium. Slides were kept at +4°C until imaging. On a separate set of specimens from peripheral retinal locations multiple fluorescent immunohistochemical reactions were carried out to label hRGCs (and other retinal neurons) with neurochemical marker Ca^++^-binding proteins by using the same staining protocol as specified above for the LY labeled retinal tissue. Appropriate mixtures of the following primary and secondary antibodies were used: mouse anti-connexin 35/36 1:1,000 (Merck Ltd, cat# MAB3045, clone 8F6.2), goat anti-calretinin 1:1,000, rabbit anti-calbindinDK28 1:1,000, goat anti-parvalbumin 1:1,000 (all from SWANT, Marly, Switzerland), donkey anti-mouse IgG conjugated with Alexa 488, donkey anti-goat IgG conjugated with Alexa 555 and donkey anti-rabbit IgG conjugated with DyLight 649 (all Alexa-conjugated antibodies were purchased from Life Technologies, Budapest, Hungary; Dy-Light conjugated antibody was Jackson Immuno Research, Europe, Suffolk, UK).

### Imaging, image processing

Images were captured on a confocal microscope (Zeiss LSM 780 with upright microscope Axio Imager Z1, Carl Zeiss Inc., Jena, Germany) using the ZEN 2012 software (Carl Zeiss Inc., Jena, Germany) and 40x Plan-Apochromat oil-immersion lens (NA: 1.4). Final images were constructed using Adobe Photoshop 7.0 (San Diego, CA, USA). Only minor adjustments of brightness and contrast were applied, which in no case altered the original appearance of the images.

### Neurolucida reconstruction and statistical analysis

Datasets were obtained from Z-stacks of flat-mount human retinal samples. Cell bodies and dendritic branches of 47 RGCs were manually traced using Neurolucida (Version 9, MBF Bioscience Europe, Magdeburg, Germany). Colocalizing Cx36 plaques were marked along the dendritic tree. Data of colocalizing Cx36 plaques were primarily obtained from NeuroLucida reconstructions and utilized for further analysis of this study. For further visualization of hRGCs we traced the dendrites using Fiji's (Schindelin et al., 2012) simple neurite tracer plugin (Longair et al., 2011). In order to obtain the correct dendrite length, the brightness and contrast were adjusted and after completion of the traces, using the fill-out option, we created new image stacks for the reconstruction. With the help of the 3D viewer plugin (Schmid et al., 2010) we created an stl (ASCII) file from the surfaces. The final videos that can be seen in our supplemental 3D material were created using Blender (Blender—a 3D modeling and rendering package, Blender institute, Amsterdam, 2018) from the surface reconstructions.

Analyses, graphical representations and statistical tests (Mann–Whitney *U*-test, Wilcoxon signed-rank test, Kolmogorov-Smirnov test, Kruskal–Wallis H test) of the results were performed by using OriginPro 2018 (Microcal Origin, Northampton, USA). Results of statistical tests were considered significant when *p* < 0.05 occurred.

We also estimated the probability density function (*P*(*x*)) of the Cx36 plaque number density with a weighted sum of log-normal components:

$$\begin{array}{l} P\left(x\right)=\overset{K}{\underset{k=1}{\sum }}\pi_{k}\cdot p_{k}\left(x\right) \end{array}$$

where *K* is the number of components and π_*k*_ is the mixing coefficient of the k-th component. The coefficients are normalized, that is:

$$\begin{array}{l} \overset{K}{\underset{k=1}{\sum }}\pi_{k}=1 \end{array}$$

*p*_*k*_(*x*) are the log-normal components which are given by:

$$\begin{array}{l} p\left(x\right)=\frac{1}{x\sigma_{k}\sqrt{2\pi }}\cdot e^{-\frac{{\left(\ln\left(x\right)-\mu_{k}\right)}^{2}}{2\sigma_{k}^{2}}} \end{array}$$

where μ and σ are the location and the shape parameters of the distribution, respectively. Assuming independent and identically distributed data we fitted the model through maximization of the likelihood given the model using an iterative procedure called the Expectation Maximization (EM) algorithm by utilizing the python scikit-learn package (Pedregosa et al., 2011). The likelihood is the probability of the data set given the parameters:

$$\begin{array}{l} L=P\left(Data|\pi ,\mu ,\sigma \right)=\overset{N}{\underset{n=1}{\prod }}P\left(x_{n}\right) \end{array}$$

The data set is the co-occurrence of observed data points (*x*_*n*_) so the probability of the data set is the product of the N individual data-point probabilities, given independent identically distributed sample. We initialized the parameters randomly and then waited 100 iteration steps, this procedure was repeated 300 times and the solution with maximal likelihood was accepted. We performed the above procedure for a different number of components (from 1 to 6). The optimal number of components (K^*^) was chosen according to the Bayesian Information Criterion (BIC). BIC shows the optimality of a model by introducing a tradeoff between the likelihood and model complexity:

$$\begin{array}{l} BIC\left(K\right)=-2\cdot ln\left(L\right)+\left(3K-1\right)\cdot ln\left(N\right) \end{array}$$

The first term on the right hand side is the negative logarithm of the likelihood, which is a monotonically decreasing function of K. In the second term is the number of free fitted parameters, namely the log-scale, the shape and the mixing coefficient for each component and minus one due to the normalization restriction for mixing coefficients. This latter term is a monotonically increasing function of K, acting as a penalty for the number of parameters, which is a simple estimate of model complexity. The optimal model has the smallest BIC value.

## Results

**Figure 1:**
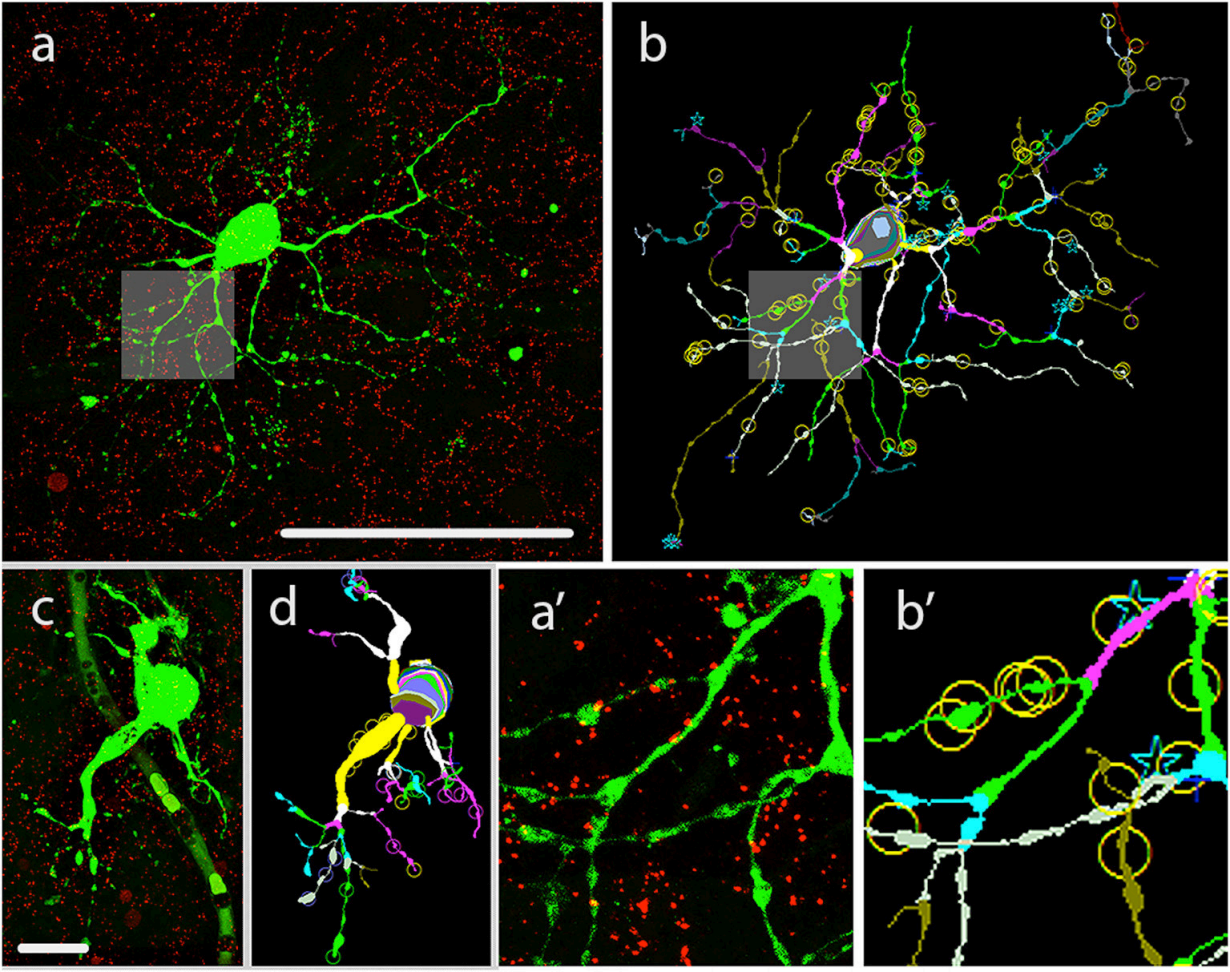
LY injection, visualization and morphometric analysis of hRGCs**. (a)** Dual stained specimen in the human retina, displays a LY injected hRGC (green) with M cell-like soma-dendritic morphology and Cx36 plaque counter labels (red). Shadings in this image and also in **(b)** mark areas enlarged in insets **(a**′**,b**′**)**. **(b)** The NeuroLucida reconstruction of the cell in panel *a* depicts the dendritic structure by marking same order dendritic segments with uniform colors. This reconstruction also points out the locations of Cx36 plaques (circles,stars over the dendrites) that were found in colocalization with a dendritic segment of the LY labeled hRGC. **(a**′**,b**′**)** Insets display enlarged area of the LY labeled cell in **(a**′**,b**′**)**. **(c,d)** LY injected small hRGC with P cell-like morphology **(c)** and the Neurolucida reconstruction of the same cell **(d)**. Scale bar: 100 μm.

Following fixation of retinal tissues hRGC somata (*n* = 47) were visualized under DIC in the whole-mount preparation and injected (see Methods) by utilizing LY as a tracer. LY diffused to all dendritic segments, thus allowed for the visualization of the entire arbor of each injected hRGC. All specimens were counterstained with an a-Cx36 serum to detect gap junction sites formed by Cx36 subunits. The LY/Cx36 dual stained materials were processed for NeuroLucida reconstruction (see section Materials and Methods) of both LY labeled hRGCs and the overlapping Cx36 plaques and used to determine colocalizations of the two labels (Figure 1). NeuroLucida reconstructions provided 3D views of the injected hRGCs and also allowed for rotation via three angles to localize Cx36 plaques precisely along the labeled dendritic branches. All colocalizing Cx36 plaques for every hRGC of this study were picked manually. This process, though tedious, allowed for the exclusion of background-, blood vessels, LY leakage and other staining that potentially introduce noise into our data. To further minimize false positive colocalizations due to the spatial resolution limit along the z-axis only Cx36 plaques whose labeling intensity maximums overlapped with those of the LY injected processes were considered. This latter process also minimized false positives due to the thicker appearance of dye filled fluorescent processes. We also rejected plaque-like structures (likely noise) that were represented by <2 × 2 pixels and/or appeared only in one optical section. Resultant morphometric data were then selected to describe the numbers, somatic proximity, dendritic distribution of Cx36 plaques as well as their tendency to aggregate across hRGC dendritic branches.

### Cx36 plaque expression by human retinal ganglion cells

**Figure 2:**
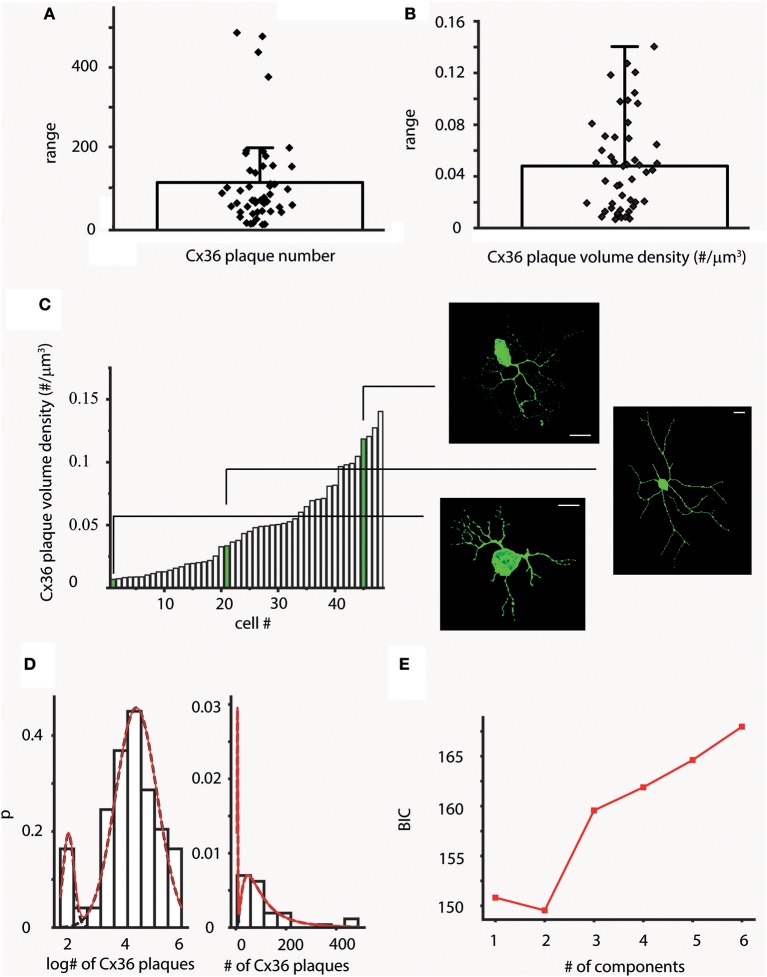
Variations in Cx36 plaque number across the hRGC population. **(A,B)** Bar-overlap charts and scatterplot showing the average values **(A)** and density **(B)** of plaque numbers colocalized with LY labeled hRGC dendrites. Overlapping scatterplot (diamonds) represents individual number **(A)** and density **(B)** values for each examined cell. The wide range of plaque counts and densities are resulted by the heterogeneous nature of the sample. **(C)** After sorting volume density values (# of plaques/μm^3^) in an ascending order the resultant histogram displays local plateaus, suggesting that several subgroups of cells with similar plaque densities exist in our dataset (see also Supplemental Figure 2 for length and surface density values). Snapshots of 3D reconstructed hRGCs corresponding to each of the subgroups are shown on the right (relevant density values are highlighted in green). **(D,E)** Forty-seven cells were clustered into two groups based on CX36 plaque numbers on dendritic surfaces. The two clusters were modeled with lognormal distributions (note the logarithmic scale), with mean plaque numbers 6.824 and 88.481; BIC minimum was determined as 2 components. Approximately 90% of the cells belonged to the cluster with the higher mean Cx36 plaque number. Scale bars: 25 mm.

GJ mediated signals from multiple sources may exert a greater effect on hRGC activity when they are efficiently summated, thus the number of functional GJ sites likely correlate with the efficacy by which GJ mediated signals contribute to the hRGC output. To examine the numerosity of such potential current sources over hRGC dendritic trees a morphometric analysis was performed to determine the total number of Cx36 plaques for each hRGC of this study. We found that all reconstructed cells displayed some Cx36 plaques over their dendritic processes. The numbers of these colocalizing plaques were rather high for most but a few cells (mean = 110.85, *SD* = ±115.63) and varied in a rather wide range (5–487; see also Figure 2A and Supplemental Table 1). This was not surprising as the sample contained hRGCs with various dendritic arbor sizes and morphologies, indicative of the heterogeneity of examined neurons. It was beyond the scope of this study to perform a thorough morphological characterization of hRGCs in the sample. However, based on the available morphological descriptions of human retinal cells (Dacey and Petersen, 1992; Kolb et al., 1992), we found parasol like (P-like) hRGCs with small soma and short, relatively simple dendritic arbors (Figures 1c,d). Dendrites of these latter cells maintained relative low number of colocalizing Cx36 plaques (Supplemental Figure 1), indicating that the observed variation in plaque counts is subtype specific.

In addition to heterogeneity, hRGCs of the same subtype with slightly different eccentricity can differ in dendritic arbor size and thus to contribute to the variation in Cx36 plaque numbers. Thus, in order to minimize these effects, the density of Cx36 plaques (densCx36) were calculated for each hRGC by dividing total Cx36 counts by the corresponding volume of the dendritic tree. The average plaque density was 0.048 plaque/μm^3^ (*SD* = ±0.037) and the actual densCx36 values covered a wide range of 0.007–0.14 plaque/μm^3^ (Figure 2B). P-like cells were among those displaying the lowest plaque densities (Supplemental Figure 1). However, when densCx36 values were plotted in an ascending order the histogram displayed multiple plateaus. This finding suggested that our sample contained several subpopulations that differed (besides the absolute numbers of Cx36 plaques) in the overall plaque density over their corresponding dendritic arbors (Figure 2C). Note, that calculations to obtain length and surface density of Cx36 plaques for the entire hRGC population were carried out as well and those provided similar results (Supplemental Figure 2).

In an attempt to separate ganglion cell subpopulations, probability density distributions of Cx36 numbers were also calculated (see methods for relevant equations). The probability density distribution showed a sign of bimodality and the subsequent mathematical model resulted in a minimum Bayesian Information Criterion (BIC) value at 2 components (Figures 2D,E). Thus, this, supporting our above morphometric analysis, further suggested the separation of cells in our sample into two populations based on the numbers of colocalizing Cx36 plaques, one with only a few plaques (mean = 6.82) and another more populous group (~90% of the cells in the sample) with considerably higher number of Cx36 plaques (mean = 88.49).

### Cx36 plaque proximity to human ganglion cell somata

**Figure 3:**
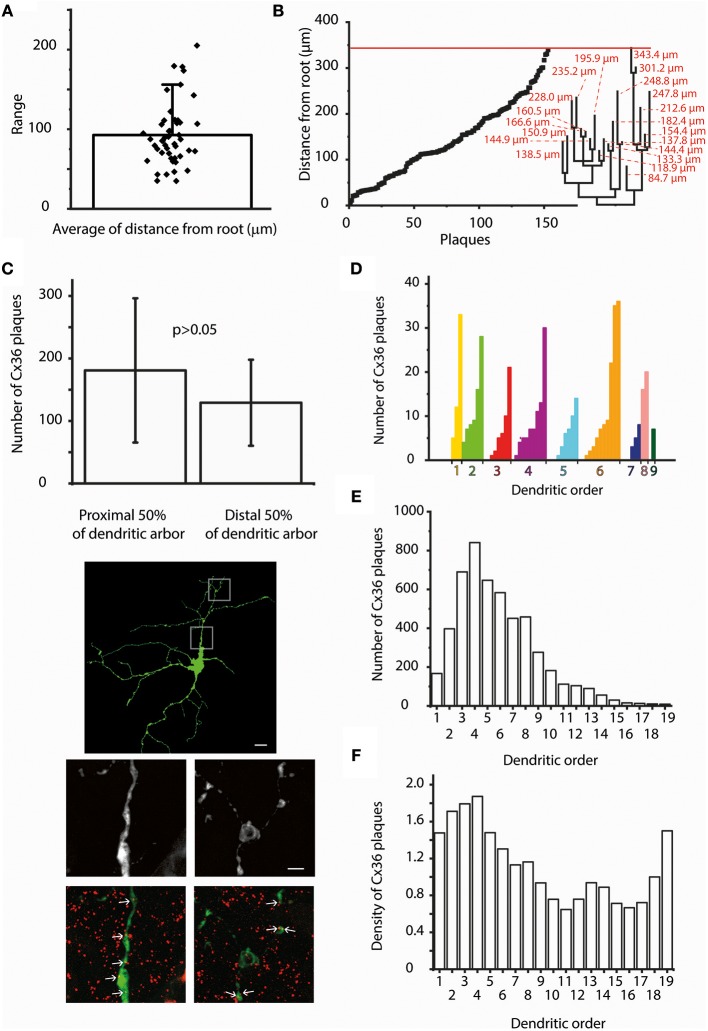
The distance from the soma and dendritic order do not influence Cx36 plaque locations. **(A)** Bar-overlap chart and scatterplot showing the average soma-plaque distance for the 47 hRGCs as well as the average distances for the individual hRGCs. It appears that the actual position of the Cx36 GJs along the dendritic arbor display a relatively even distribution and also a rather wide range. **(B)** Soma-plaque distances were plotted in an ascending order for a representative hRGC. It appears that values display an overall even distribution. Note, that neighboring values do not necessarily correspond to nearby plaques on the same segment or even on the same dendritic branch. Dendrogram on the right shows the structure of the entire arbor with soma-tip distances (red). Red line on the top delineates the distance of the distalmost terminal ending. This combined image shows that distal dendritic locations are just as populous as proximal ones, thus the soma-plaque distance most likely is not a principal factor in determining Cx36 plaque locations. **(C)** Bargraph compares the average Cx36 plaque numbers within the proximal and distal half arbors for six randomly selected hRGCs with large dendritic trees (bars over the columns represent SDs). Clearly, the amount of Cx36 are not significantly different between the proximal and distal halves of dendritic arbors (Wilcoxon signed-rank test, *p* = 0.22). Photomicrographs show a representative hRGC with large dendritic field (top). High magnification images show details of the proximal (middle left) and the distal (middle right) dendrites; images on the bottom show corresponding LY/Cx36 double labeled versions of the same dendritic areas. Largely the same number of Cx36 colocalizations (arrows) could be recognized in both proximal and distal dendritic arbors. **(D)** Histogram displays the number of colocalizing Cx36 plaques for each dendritic segment of a representative hRGC. Dendritic segments are grouped based on their orders and then sorted into an ascending order of the colocalizing Cx36 numbers. This analysis showed no obvious sign of order related preference for the location of plaques as segments with both low and high Cx36 numbers were found for most branch types. **(E)** Histogram displays the Cx36 plaque numbers for all segments of all examined cells of this study as a function of the segmental dendritic order. This analysis resulted a lognormal distribution of the colocalizing plaques with the highest values in 4th order dendrites. **(F)** In order to account for the difference in numbers of segments within a certain dendritic order, the plaque density was calculated and plotted against the dendritic order for all segments of all examined cells. The highest amount still found in the 4th order dendrites, but with a more homogenous distribution. In addition, this analysis revealed a second peak of the histogram for the highest order processes suggesting that terminal dendritic segments are preferred locations for Cx36 plaques. Scale bars: 25 and 5 μm in the low- and high magnification panels, respectively.

Signal efficacy might also be determined by the distance that GJ mediated currents must travel from their origin (GJ site) to the cell body where incoming information is integrated to generate the spike output toward the brain. Therefore, a second set of analyses was carried out to determine the average distance of Cx36 plaques to the soma for all hRGCs. The values covered a wide range of 35–205.1 μm, and the total average was 92.2 μm (SD ±39.1; Figure 3A; Supplementary Table 1). Due to this wide range it is unlikely that distance is a determining factor in the Cx36 plaque distribution over hRGC dendritic arbors.

To further examine this issue soma-plaque distances were plotted in an ascending order for hRGCs. Cx36 plaque distance values appeared to display even distributions for most examined cells (Figure 3B). This finding was supported also by our analysis showing that hRGCs with relative large arbors (*n* = 6) had just as many plaques in distant dendrites as in proximal ones (Figures 3B,C; Wilcoxon signed-rank test, *p* = 0.22). Overall, the above findings indicated that distal dendritic locations were just as populous as proximal ones, thus the soma-plaque distance most likely is not a principal factor in determining Cx36 plaque locations.

To gain more insight into Cx36 plaque distribution we performed an analysis that rendered Cx36 plaque numbers to the order of their corresponding dendrites (Figures 3D–F). The orderly distribution of plaques varied considerably as shown by the diversity of the obtained distribution histograms and no obvious general features were seen. The great variety in this analysis was not surprising as our heterogeneous sample contained dendritic arbors with branches of 4–19th orders. This indicated that Cx36 plaque distribution was largely independent of dendritic order.

**Figure 4:**
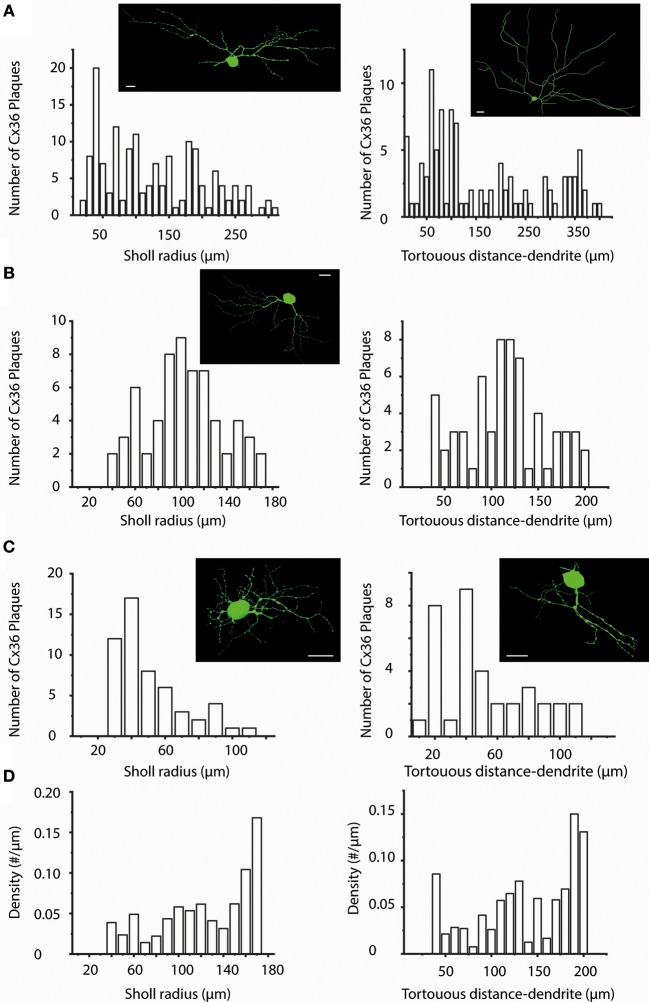
Sholl- and Tortuous Distance Analyses of Cx36 plaques over hRGC dendritic arbors. **(A–C)** Sholl (left panels) and Tortuous Distance (right panels) analyses shown for representative hRGCs (top, middle, and bottom) to visualize the Cx36 plaque distribution as a function of distance (10 μm bins) to the soma. As our sample contained many different hRGC subtypes we found plaque distributions with very different patterns including those appeared random (**A,C** right), showing near Gaussian shape **(B)**, descending (**C** left). These analyses thus did not seem to reveal preferred dendritic locations for Cx36 plaques that are general features for the entire examine hRGC population. Images on the top of each panel display a representative hRGC. Note, that both left and right panels in b display histograms of the same hRGC. **(D)** Due to the fact that actual dendritic segment numbers and lengths may differ considerably in each step window, the plaque density was also plotted against both the Sholl (left) and the Tortuous Distance (right) radii. The two panels in **(D)** shown here were generated on the dataset for the hRGC shown in **(B)**. These graphs clearly show in both the Sholl and the Tortuous Distance Analyses that the distalmost bins display the highest plaque density, suggesting that dendritic terminal endings bear a special importance. Scale bars: 25 μm.

Next, both a “Sholl” and a “Tortuous distance” analyses with 10 μm step-size were carried out in all cells of the sample. The obtained histograms varied considerably in both cases. The plaque distribution pattern seemed random for many cells, showed near Gaussian distribution for others or appeared linearly descending (Figures 4A–C). However, results of this analysis were highly affected by the number of processes falling into each step-window. This was especially true for hRGCs where the middle area of the arbor comprised significantly higher number of processes than the proximal or the distalmost areas, in which cases both the “Sholl” and “Tortuous distance” analyses resulted in a near Gaussian distribution. To correct for this the analyses were repeated by calculating the number of Cx36 plaques over a unit length (μm) of the dendritic arbor. These repeated analyses changed the appearance of histograms into more uniform and most cells now showed a near linear plaque distribution over the dendritic arbor with no Gaussian like histograms. However, the most dramatic finding was that numerous cells (*n* = 28) displayed the highest relative plaque numbers in their corresponding highest bins. This suggested that the dendritic endings or terminal branches tend to maintain a more numerous Cx36 plaque population than other dendritic areas (Figure 4D).

### Cx36 plaque distribution across dendritic arbors of human ganglion cells

**Figure 5:**
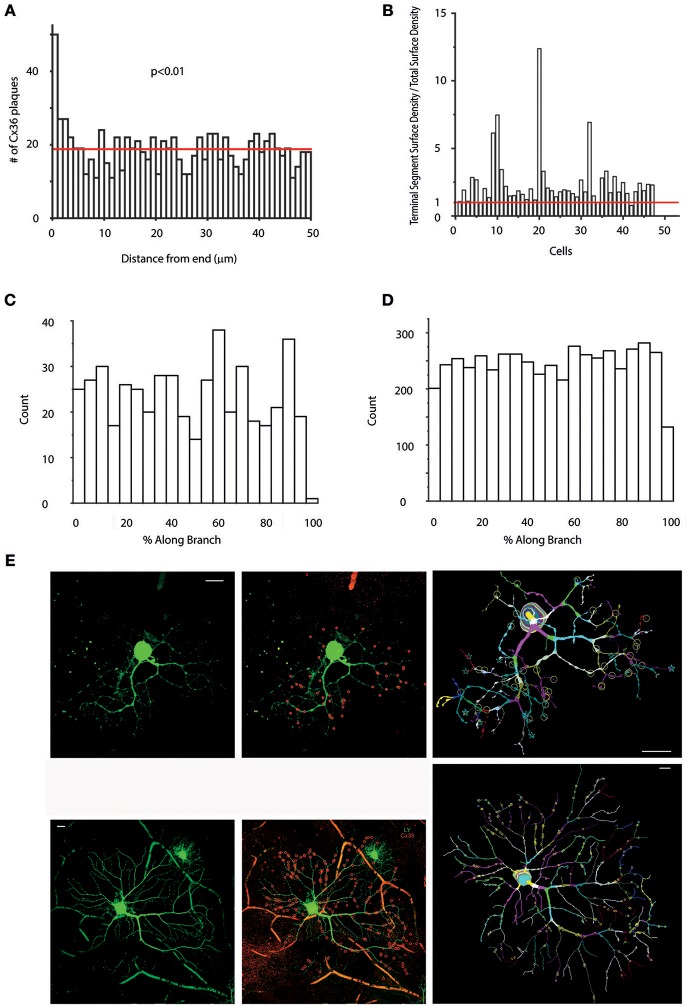
Preferred Cx36 plaque locations in hRGC dendritic arbors. **(A)** Histogram showing the numbers of Cx36 plaques located at only terminal segments as a function of the distance from the ending. Note, that only terminal segments with lengths >50 μm (shorter segments would skew the analysis toward the lower values) were selected for this analysis (the red line marks the mean). Clearly Cx36 plaques located at terminal dendritic segments prefer sites that are close to the very endings (Wilcoxon signed-rank test). **(B)** Histogram shows the “Terminal Segment Surface Density/Total Surface Density” ratio for cells in this study (x axis) to reveal if terminal endings are preferred sites for Cx36 plaques. It can clearly be seen that the terminal segment density is higher than the overall density (red line) in case of most (44 out of 47) cells (see also length and volume density charts in Supplemental Figure 3). **(C,D)** Graphs display Cx36 plaque counts as a function of the percentage of total length (distance from proximal branch-point/total segment length *100) of their respective dendritic segment for one representative hRGC **(C)** and all cells in the sample **(D)**. Note, that these analyses contain only non-terminal segments, whereas previous analyses in **(B)** examined only terminal segments. Cx36 plaques with lower values are located in the vicinity of proximal end of the segment (end closer to the soma) whereas Cx36 plaques with high values are close to distal branch points (end farther from the soma). We found that both individual histograms and the combined histogram displays no sign of any favored segment locations for Cx36 plaques. **(E)** Two representative hRGCs (left panels) maintaining many Cx36 plaques (red circles in the middle panels and letter marks in the right panel; red circles mark only colocalized Cx36 plaques that were confirmed in Neurolucida reconstructions) over their dendritic arbor. Many colocalizing Cx36 plaques were located near to the terminal endings as shown in both the photograph (middle) and the corresponding Neurolucida reconstruction (right) for both neurons. The cell on the bottom displays a soma-dendritic morphology reminiscent of parasol (M) hRGCs based on previous descriptions by Kolb et al. (1992) Scale bars: 25 μm.

It appeared in the previous analyses that terminal dendritic branches maintained a relative high number of Cx36 plaques for most cells in our sample. In order to gain a deeper insight in this issue first we examined all hRGCs in our sample (*n* = 47), to see if Cx36 plaques at the very end of dendrite terminals were in higher numbers relative to those with higher plaque-to-end distances. We found that the Cx36 number was considerably higher at < 1 μm distance from terminal endings than more proximal terminal segment areas and this difference was significant (Figure 5A; Wilcoxon signed-rank test, *p* < 0.01).

To examine this issue further, we next obtained dendrograms for all hRGCs, and Cx36 plaque numbers for both terminal dendrites and entire dendritic arbors were collected. Next, we performed a “Terminal segment analysis” by which the “terminal segment plaques/total plaque number” and the “terminal segment length/total dendrite length” ratios were utilized to determine “terminal segment density/total dendritic density” rates for all hRGCs (Figure 5B, Supplemental Figure 3). We found that density rates of most hRGCs (*n* = 42 out of 47; ~89%) were >1 indicating higher plaque density over terminal branches (Figure 5B); Supplemental Figures 3A,B). Moreover, the density rate was >2 for almost a quarter of cells (*n* = 11 out of 47; ~23%) suggesting that Cx36 plaques were twice as numerous at terminal dendrites of these cells than over the rest of their arbors, and for some cells (*n* = 4 out of 47; ~8%) the density rate was 3–7 times higher. These numbers clearly showed that the terminal dendritic branches of hRGCs are preferred sites to form gap junctions with other inner retinal neurons Figure 5E.

The dendritic distribution and location of Cx36 plaques were further examined by obtaining information on the position of Cx36 plaques in each dendritic segment with regard to the vicinity of dendritic branches. Calculating the Cx36 plaque distance along branch as a fraction of the total segment length allowed the observation and comparison of plaque positions on their respective segments. In this analysis, plaques with relative low and high values were located in the vicinity to proximal (closer to the soma) and distal (farther from the soma) ends of the segments, respectively. In contrast, the rest of the plaques were located in the midst of their corresponding segments. The frequencies of these distance fractions per cell were plotted. Counting the number of plaques within the first and last 10% of each segment, gave an indication whether the preferred plaque position was closer to either ends of the segments. These analyses were performed for every cell and no general pattern could be defined suggesting that the vicinity of dendritic branches is not a general plaque location determining factor (Figures 5C,D).

### Tendency of cx36 plaques to form clusters

**Figure 6:**
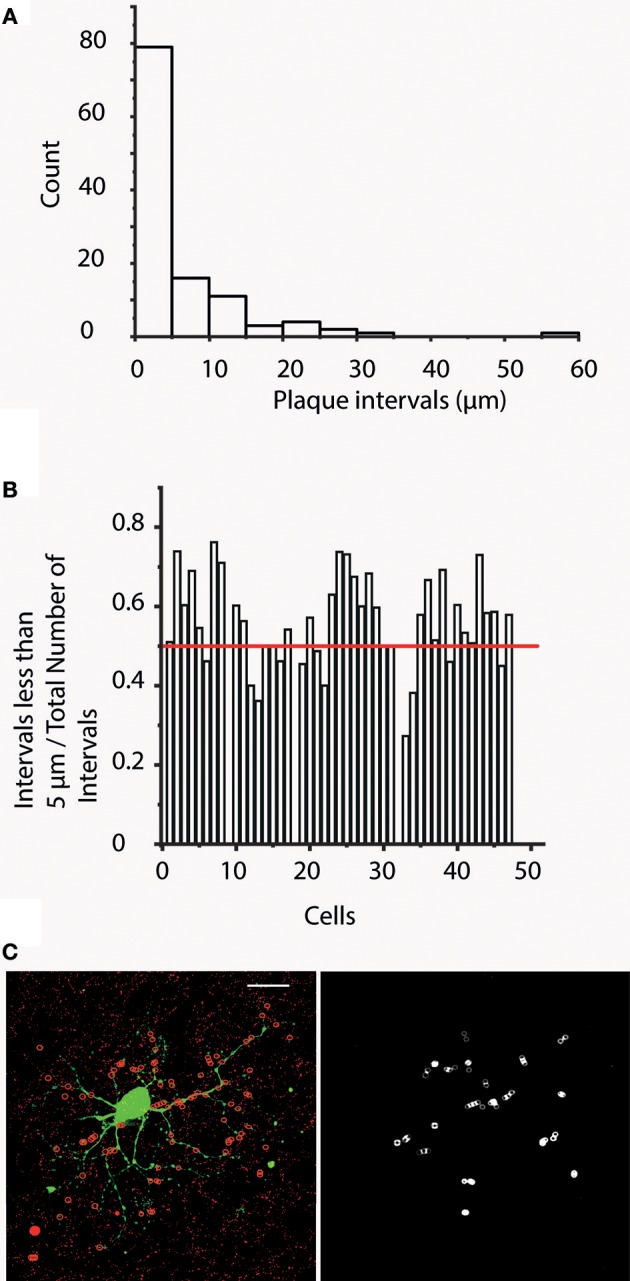
Cx36 plaques tend to form clusters. **(A)** Bar histogram shows the results of “plaque interval” measurements (bin size 5 μm) for one representative hRGC. This analysis provides distance values measured between each plaque and its direct proximal neighbor. It is clear that plaque distances < / = 5 μm occur most often indicating that Cx36 plaques of this neuron have a tendency to cluster into doublets or perhaps even triplets. **(B)** This histogram displays the ratio of the number of < 5 μm intervals and the total number of intervals for all hRGCs of this study. This analysis indicates that 33 cells display ratios >0.5, suggesting that the majority of the plaques for these 33 cells form pairs/clusters. Note, that very small cells with only one plaque per segment have no plaque intervals, hence on this chart they show up with a value of 0. **(C)** Image on the left displays a representative hRGC and Cx36 labels. Colocalizing plaques were marked with red circles overlaid on dendrites for better visibility (red circles mark only colocalized Cx36 plaques that were confirmed in Neurolucida reconstructions). Note that this hRGC is the same as the one shown in Figure 1. The image to the right display circles that mark direct neighbor Cx36 plaques in < 5 mm apart. Scale bars: 25 μm.

Having obtained the aforementioned dendrogram for each cell, a “plaque distribution on segment” analysis was also performed and plaque distances measured from the beginning of each segment were recorded (“distance along branch”). These measurements provided data on distances between plaques allowing for the generation of “distance frequency” histograms for each hRGC. This analysis showed that a high percentage of plaque intervals (1866 out of 3390) were 5 μm or less (Figure 6A). To support this observation, ratios of nearby (< 5 μm) and total plaque numbers on each dendritic segments were calculated. This analysis showed that 33 out of 47 cells had ratios >0.5 meaning that more than half of the examined plaques were closer than 5 μm to their nearest neighbor (Figure 6B and C). This indicated that a significant portion of plaques formed clusters (at least pairs) for most examined hRGCs. Furthermore, we counted 2.95 times as many plaques as plaque bearing segments suggesting that many segments had more than one (~3) plaque on their surfaces (*n* = 3,227 out of 3,990 segments in all of the cells; 80.9%).

In order to analyse the distribution of Cx36 plaques on unique segments, we performed Kolmogorov-Smirnov test on each Cx36 bearing segment with at least 10 samples against uniform density of plaques and also against exponentional density of plaque distance intervals. No significant (*p* < 0.01) deviation from uniform density or exponential density were detected (Bonferroni correction was applied). We also performed a pooled analysis of Cx36 plaque densities over all segments to shed light on Cx36 density inhomogeneities. We calculated plaque densities up to 140 μm distance along segments with 20 μm bin resolution for each segment, and treated the calculated density values as separate populations per bin. We compared the population means (Kruskal-Wallis H test) and found that the mean densities over bins significantly deviated from uniform density (*p* < 0.01). This analysis showed that Cx36 plaque density was not uniform over segment length, however the small number of Cx36 plaques per unique segments prevented drawing straightforward conclusions about the spatial distribution-pattern of plaques.

## Discussion

### Methodological considerations

Due to the obvious difficulty obtaining human material for experimental purposes, the quantity of our specimen was limited in terms of the utilized samples (*n* = 3 individuals; see Materials and Methods). In addition, only a fraction of the eyes was available for us to inject and counter label hRGCs. Another limiting factor was the challenging nature of the successful intracellular injection of cells in the fixed post-mortem retinas. However, we still successfully injected hRGCs with a rather high success rate (~60–70%) in such material by adopting and refining a previously existing method (Kántor et al. 2016b). The pool of injected cells contained over 80 hRGCs but only 47 cells with fully labeled arbor and good quality Cx36 counterlabels were used in this work. Our goal was to label hRGCs with various soma sizes to obtain data for several (preferentially all) hRGC subtypes in the human retina. However, certain cell types were easier to encounter and inject thus they are likely overrepresented in our sample. Some of the well-represented hRGCs resemble P or M cells based on their morphological characteristics (Kolb et al., 1992), whereas others may have only a few (or none) representatives in our sample. One hRGC type, with very large cuboid soma were targeted several times but failed to fill fully with the LY dye. We suspect that this cell corresponds to the G23 (monster) cell in Kolb's dataset. This was supported by the fact that we lacked a cell in our sample that shared the characteristic morphological features of G23 cells including the largest somata in the human retina (Kolb et al., 1992). Another limitation of our dataset was that all utilized tissues originated from peripheral areas, which prevented us to examine possible area specific variations in the plaque distribution of hRGCs. However, such area specific comparison is out of the focus of this study and the limited area specific sampling also lowers the number of variables that can affect the dendritic distribution of Cx36 plaques. Contrary to all the above listed limitations this study provides the first overall view on the dendritic distribution of Cx36 plaques over hRGC arbors. Such description allows us to further assess the degree of similarity (or dissimilarity) of hRGCs and those of model animals in order to infer and use results of animal studies for clinical purposes.

### Cx36 plaque labels of hRGCs

We reported previously that certain Ca^++^-binding proteins including calretinin (CaR), parvalbumin (PV) and calbindin (CaB) are expressed (exclusively or in combination) in specific subsets of hRGCs (Kántor et al., 2016b). Although, we found colocalizations of Cx36 plaques with these hRGC markers (Supplemental Figure 4) these markers were not selective but labeled multiple cell types and many of those were non-hRGCs. Therefore, multiple label immunohistochemistry was not an adequate method to collect data for this present study. In contrast intracellular LY injections in the fixed human sample appeared to provide hRGC labels in sufficient quality that allowed us to collect data.

RGCs of many animal models maintain GJs with neighbor cells and most of these contacts are Cx36 dependent (Hidaka et al., 2004; Schubert et al., 2005a; Völgyi et al., 2005, 2009; Pan et al., 2010). Therefore, it is not surprising that dendritic branches of many cells in our sample colocalized with Cx36 plaques in the counterstained specimen. Although, each potentially colocalizing plaque was thoroughly tested it is inevitable that few colocalizations are only virtual and added noise to our dataset. It is possible to estimate the signal to noise ratio by examining Cx36 colocalizations of hRGCs for cells that have been confirmed uncoupled and thus do not maintain GJs. However, no reports have been published on hRGC GJ coupling and Cx36 expression yet. On the other hand, non-human primate P-cells (parvocellular ganglion cells) have been shown uncoupled when GJ permeable tracer was injected into their soma, whereas M cells maintained extensive tracer couplings (Dacey and Brace, 1992). Assuming that non-human primate and human retinas are similar in this respect, Cx36 colocalizations with human P-cells only happen by chance and not due to the existence of P cell GJ sites. We did not perform a RGC classification in this work but performed a raw sorting based on soma/dendritic dimensions and morphology of hRGCs, which resulted in P-like and non-P-like group of cells. We found in fact that cells in the P-like group associated with low number and density of colocalizing plaques. Both the plaque numbers and density values were significantly lower for P-like cells than those of non-P-like cells even though the latter group was still a heterogeneous neuron population of coupled and uncoupled cells (Supplemental Figure 1, Mann–Whitney U test; plaque number: *p* = 1.24 × 10^−4^, plaque SA Density: *p* = 0.023). This observation thus suggests that both non-human and human P cells are uncoupled and also indicates that our dataset contains a low level of noise in regard of LY/Cx36 colocalizations. Assuming the same noise level for all examined cells (P-like and non-P-like) we conclude that the signal-to-noise ratio (S/N) in this study was high enough to observe colocalizations associated with real hRGC GJ sites and draw conclusions based on collected data. In addition, apparent colocalizations of Cx36 plaques and putative uncoupled P-like hRGCs also sets the limit for false positive labels. Supplemental Figure 1 shows that only a small set of non-P cells display Cx36 numbers as low as those of P-like hRGCs. This indicates that the noise due to false positive labels of this study is rather low. It also suggests that similar to the Cx36 expression of RGCs other mammalian species (Völgyi et al., 2009; Pan et al., 2010) most hRGCs maintain Cx36 GJs to contact neighbor RGCs and/or amacrine cells.

### Strategic positioning of cx36 plaques across hRGC dendritic arbors

One of our main aims throughout this study was to identify particular locations on hRGC dendritic trees that are preferred sites for Cx36 GJs. We failed to reveal favored Cx36 plaque distance to soma, favored dendritic segment order or the preference for the vicinity to dendritic branching points. On the other hand, we have to point out that our sample is comprised by a heterogeneous neuron population thus our analyses only allowed us to reveal general features shared by the majority of the examined hRGC population. Besides these general features it is possible that certain hRGC subtypes possess preferred Cx36 GJ distance to soma, dendritic order or GJ location along dendritic segments. In fact, we expect that a larger pool of hRGCs could be subdivided based on morphological features. Such a collection of data will allow for recognizing potential cell type specific variations of GJ sites. Contrary to the heterogeneity of the dataset we found a few marked general features of Cx36 GJ locations over hRGC dendrites. These included the tendency of Cx36 plaques to position frequently on terminal dendritic branches. Moreover, we found evidence that many of these GJ sites are >5 μm from the very end of their corresponding terminals. This finding suggests that certain intercellular contacts favor the periphery of hRGC dendritic arbor areas. It has been shown that alpha RGCs in the rat retina maintain tip-to-tip GJ contacts with their immediate neighbors that allows them to form electrically coupled arrays (Hidaka et al., 2004). The existence of similar homologous RGC-to-RGC electrical and tracer coupling has been shown for many other mammalian species as well-including rabbits, mice and non-human primates (Dacey and Brace, 1992; DeVries, 1999; Schubert et al., 2005a,b; Völgyi et al., 2005, 2009, 2013a,b). Such pan-mammalian feature of RGCs is then very likely shared by at least a few subsets of hRGCs as well. Therefore, we think that the observed Cx36 plaques found in the dendritic terminals reflect sites of hRGC-to-hRGC homologous GJs. The tip-to-tip configuration is a necessary arrangement, according to which RGCs obey to territorial rules to avoid significant dendritic overlaps with same subtype neighbors thereby reducing redundant sampling and at the same time they still maintain minimal dendritic overlap that allows nearby RGC to form direct GJ contacts. Besides homologous GJs, model animal RGCs electrically couple to nearby amacrine cells as well (Kenyon and Marshak, 1998; Hu and Bloomfield, 2003; Schubert et al., 2005a; Völgyi et al., 2005, 2009, 2013a,b; Bloomfield and Völgyi, 2009; Hu et al., 2010; Pan et al., 2010; Roy et al., 2017). This latter set of GJs serve as an indirect contact between RGCs in an extended (several hundreds of μm-s) retinal surface area (Bloomfield and Völgyi, 2009; Hu et al., 2010). The fact, that amacrine cells which are involved in these heterologous GJs are typically wide-field and polyaxonal amacrine cells (Schubert et al., 2005a; Völgyi et al., 2005, 2009; Roy et al., 2017), means that their processes run several hundreds of μm-s and that they do not share the territorial avoidance rule with the contacting RGCs. Consequently, the RGC-to-amacrine cell GJs are not necessarily located on RGC terminals, but they can be formed on any RGC dendritic segment. Following this logic, the number of non-terminal Cx36 GJ sites must exceed those of terminal dendrite Cx36 GJs, because model animal RGC-to-amacrine cell GJs are maintained by most RGC subtypes, whereas RGC-to-RGC direct contacts are established by only a handful of RGC populations (Völgyi et al., 2009, 2013b; Pan et al., 2010). These observations are now supported by our finding in the human retina that the overall number of Cx36 plaques was rather high for most but a few cells in our sample with higher density values in terminal dendrites. This suggests that hRGCs, similar to their model animal counterparts, form both heterologous hRGC-to-amacrine and homologous hRGC-to-hRGC contacts. These two types of RGC coupling patterns have been shown to serve various forms of action potential synchronizations in model animals (Mastronarde, 1983; Brivanlou et al., 1998; DeVries, 1999; Hu and Bloomfield, 2003; Bloomfield and Völgyi, 2009; Völgyi et al., 2013a,b), therefore we posit that they fulfill similar spike synchronization roles for hRGCs as well. In addition, it has been shown that Golgi interneurons in the cerebellum maintain distal dendritic GJs in high densities to counteract sublinear dendritic integration and enable distal excitatory synapses to drive network activity more effectively (Vervaeke et al., 2012). Similarly, a higher density of Cx36 GJ plaques was found in hRGCs of this study suggesting that besides spike synchronization hRGC GJs at terminal endings may serve to counteract sublinear integration of chemical synaptic inputs due to passive dendrite cable properties.

### Tendency of cx36 plaques to form clusters

A second general feature of this Cx36 plaque distribution survey was the finding that plaques along dendritic segments have a tendency to cluster together. This clustering of Cx36 plaques, to the best of our knowledge, has not been shown before even in model animal experiments. Our dual staining methodology does not allow the visualization and identification of the synaptic partners of hRGCs that partake in GJ contacts, therefore we can only speculate about the function of such plaque cluster formation. If clustered GJs are formed by the same synaptic partner than it is possible that two or three contacts are formed nearby to strengthen the efficacy of the electrical synaptic connection between the contacting neurons. On the other hand, if nearby GJ sites are established with dendritic processes of two (or more) synaptic partners, then there is a possibility for the integration of their inputs. In this scheme, depending on the timing of the incoming signals and/or refractory period inputs, they can enhance or abolish each other functioning as AND, OR, or XIR logical gates, respectively. Besides the possibility of parallel inputs to hRGCs through nearby GJs, it is also possible that GJ site doublets also serve as outputs from hRGCs to multiple synaptic partners, likely amacrine cells. In this latter scenario, hRGCs (and likely RGCs of mammalian models as well) besides sending information toward visual brain centers would also serve with intraretinal output signals for the retinal hypercircuit as well. Through such information loops certain RGC subtypes may affect the functioning of other retinal RGC subtypes. However, the present dataset is very limited and thus future experiments are necessary to prove any of the above hypotheses.

## Author contributions

OK performed most experiments and helped out with the experimental design, GD performed some experiments, GS did most of the analysis and some of the manuscript writing, ZB and ZS performed some of the analysis, EP, GR, and RN helped out with the experiments, GB obtained tissues for the experiments and BV designed the experiments, did some of the analysis and wrote the manuscript.

### Conflict of interest statement

The authors declare that the research was conducted in the absence of any commercial or financial relationships that could be construed as a potential conflict of interest.

## Supplementary material

Supplemental Figure 1 Number (top) and plaque density (bottom) differences between P-like and non-P-like hRGCs. Statistical analyses reveal that P-like cells display considerably less Cx36 plaque colocalizations than non-P-like cells (Wilcoxon signed-rank test *P* < 0.05). The somata and dendritic arbors of representative P-like and non-P-like hRGCs are shown above the corresponding bars. Scale bars: 25 μm.

Supplemental Figure 2 Number **(A)**, plaque length density **(B)** and plaque surface density **(C)** histograms for all examined hRGCs display non-homogenously increasing values with several plateaus. This indicates that cells in these analyses form subgroups.

Supplemental Figure 3 **(A,B)** Terminal segment length density / total length density **(A)** as well as terminal segment volume density/total segment volume density **(B)** ratios are presented. Should a cell have value >1, that means plaques of that certain cell are most frequent in their terminal segments. Both charts display that for most cells (length density: 42 out of 47 cells, volume density: 34 out of 47) the ratios are > 1.

Supplemental Figure 4 **(A–F)** Images display multiple immunolabels of PV, CaR, CaB, and Cx36. Colocalizing Cx36 plaques can be detected on CaR+ **(A,B)**, on CaB+ **(C,D)**, on PV+ **(E)** and on CaB/PV dually stained **(F)** hRGC dendritic processes. It has been shown that PV/CaB dual labeled hRGCs display OFF parasol cell morphology (Kántor et al., 2016b). Scale bars: 20 μm.

Supplemental Table 1 Table shows measured parameters for all examined hRGCs of this study (left column), the total dendritic lengths (second column, number of all colocalizing Cx36 plaques (third column), the number of Cx36 plaques colocalizing with terminal dendrites, number of all plaque intervals for each cell (fourth column) and the number of plaque intervals < 5 micrometer.
