# Supplementary figures and images for: Strategic Positioning of Connexin36 Gap Junctions Across Human Retinal Ganglion Cell Dendritic Arbors
Source: Front Cell Neurosci. 2018 Nov 22;12:409. doi: 10.3389/fncel.2018.00409 (PMC6262005; doi:10.3389/fncel.2018.00409)

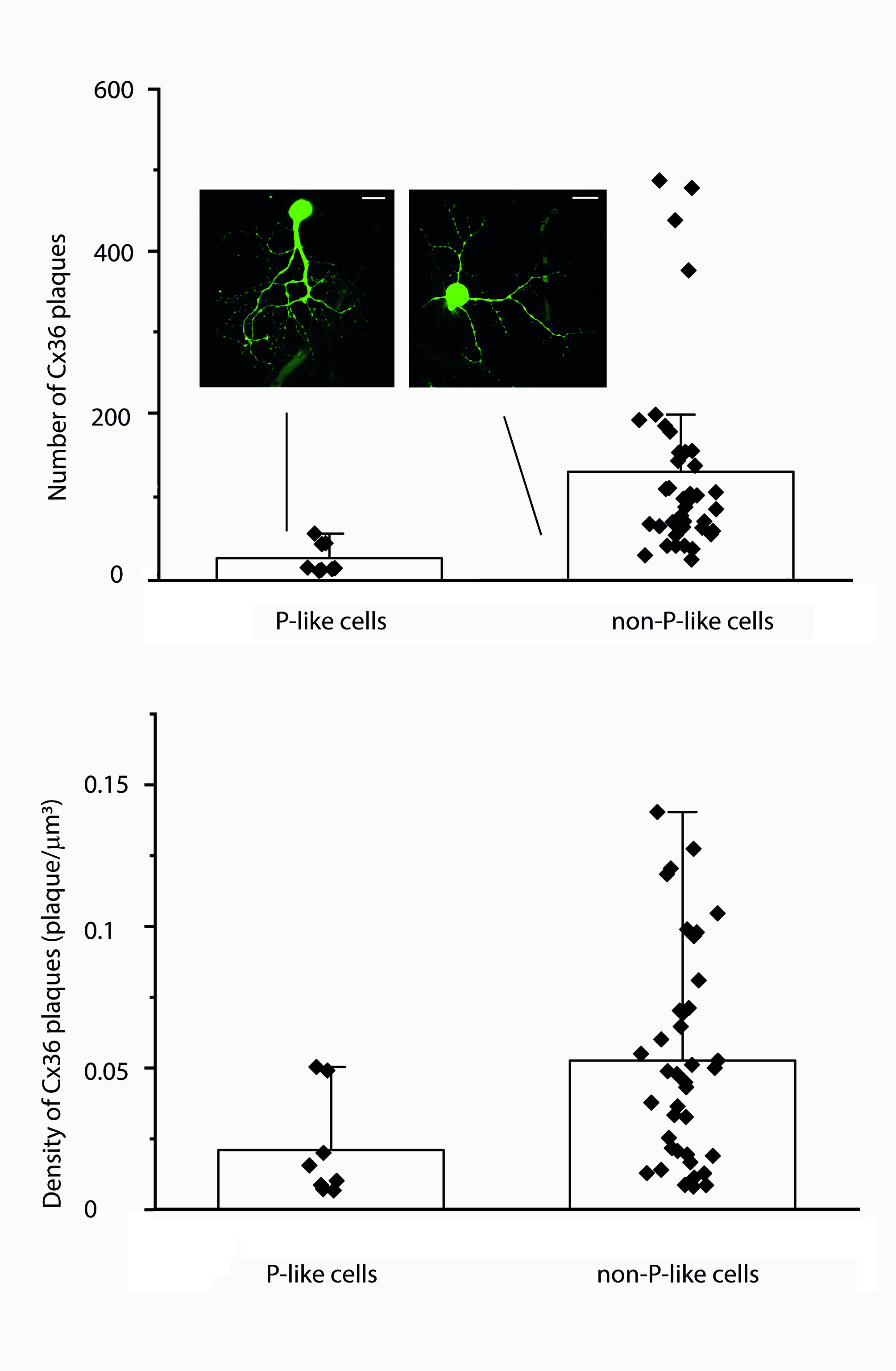

Supplement: Supplemental Figure 1 — Number (top) and plaque density (bottom) differences between P-like and non-P-like hRGCs. Statistical analyses reveal that P-like cells display considerably less Cx36 plaque colocalizations than non-P-like cells (Wilcoxon signed-rank test P < 0.05). The somata and dendritic arbors of representative P-like and non-P-like hRGCs are shown above the corresponding bars. Scale bars: 25 μm. [file Image_1.tif]

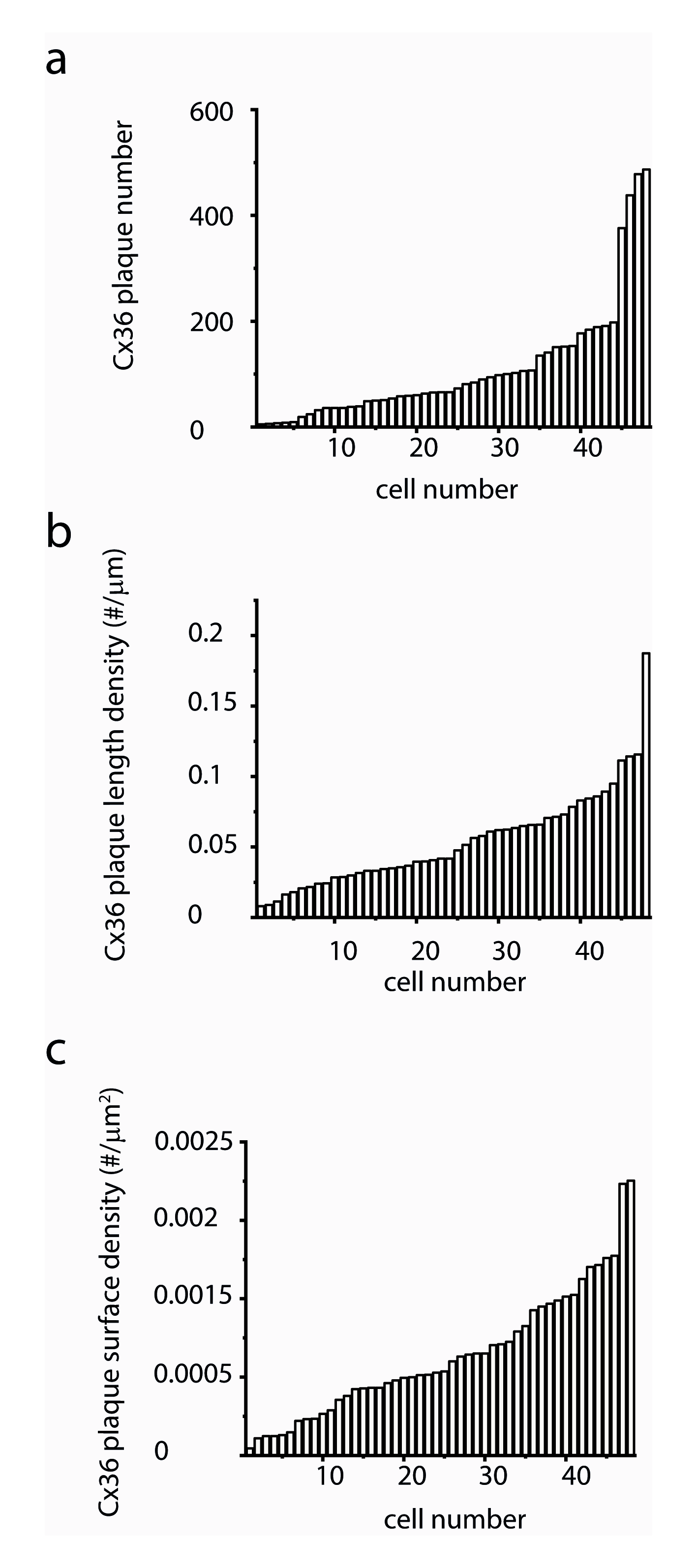

Supplement: Supplemental Figure 2 — Number (A), plaque length density (B) and plaque surface density (C) histograms for all examined hRGCs display non-homogenously increasing values with several plateaus. This indicates that cells in these analyses form subgroups. [file Image_2.tif]

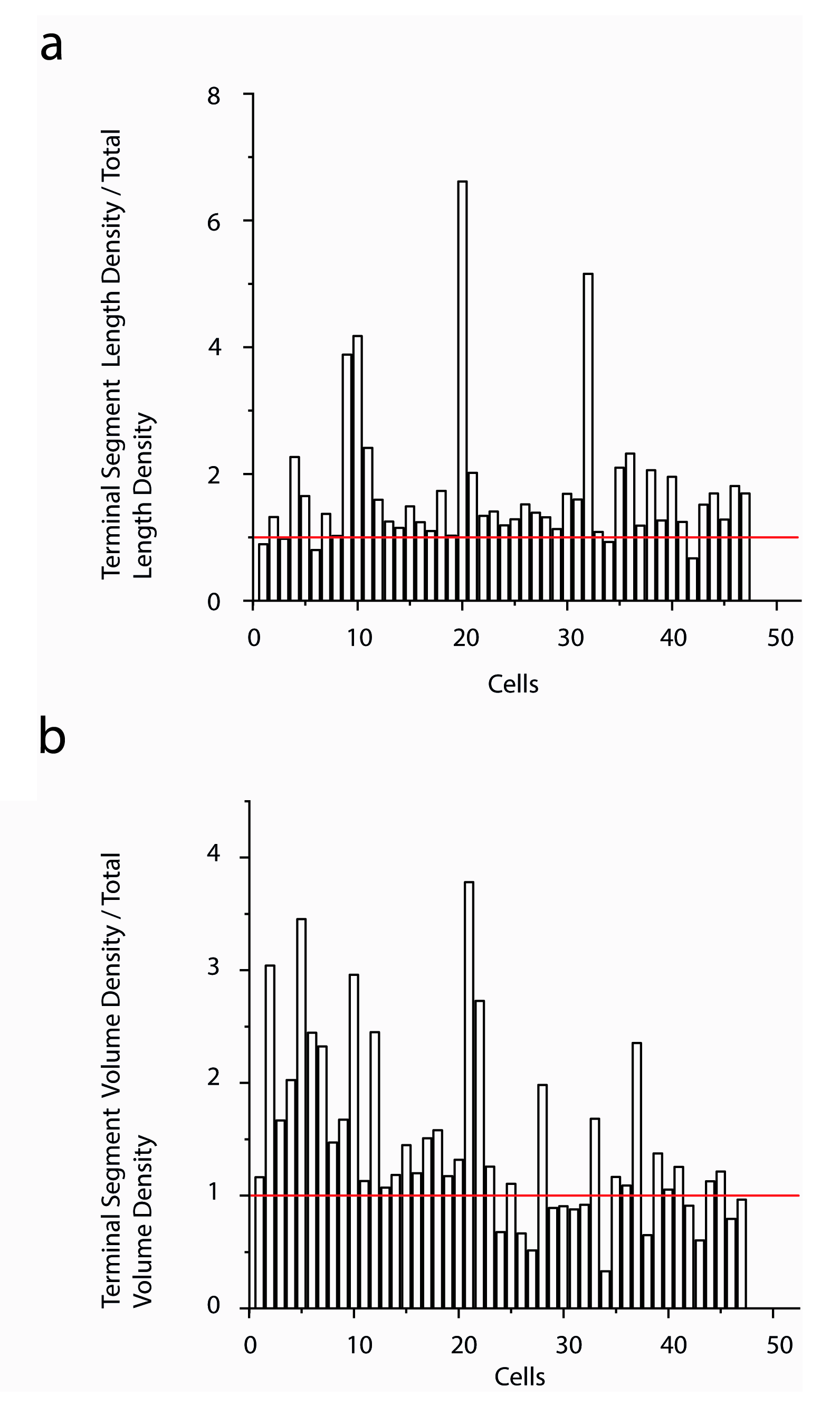

Supplement: Supplemental Figure 3 — (A,B) Terminal segment length density / total length density (A) as well as terminal segment volume density/total segment volume density (B) ratios are presented. Should a cell have value >1, that means plaques of that certain cell are most frequent in their terminal segments. Both charts display that for most cells (length density: 42 out of 47 cells, volume density: 34 out of 47) the ratios are > 1. [file Image_3.tif]

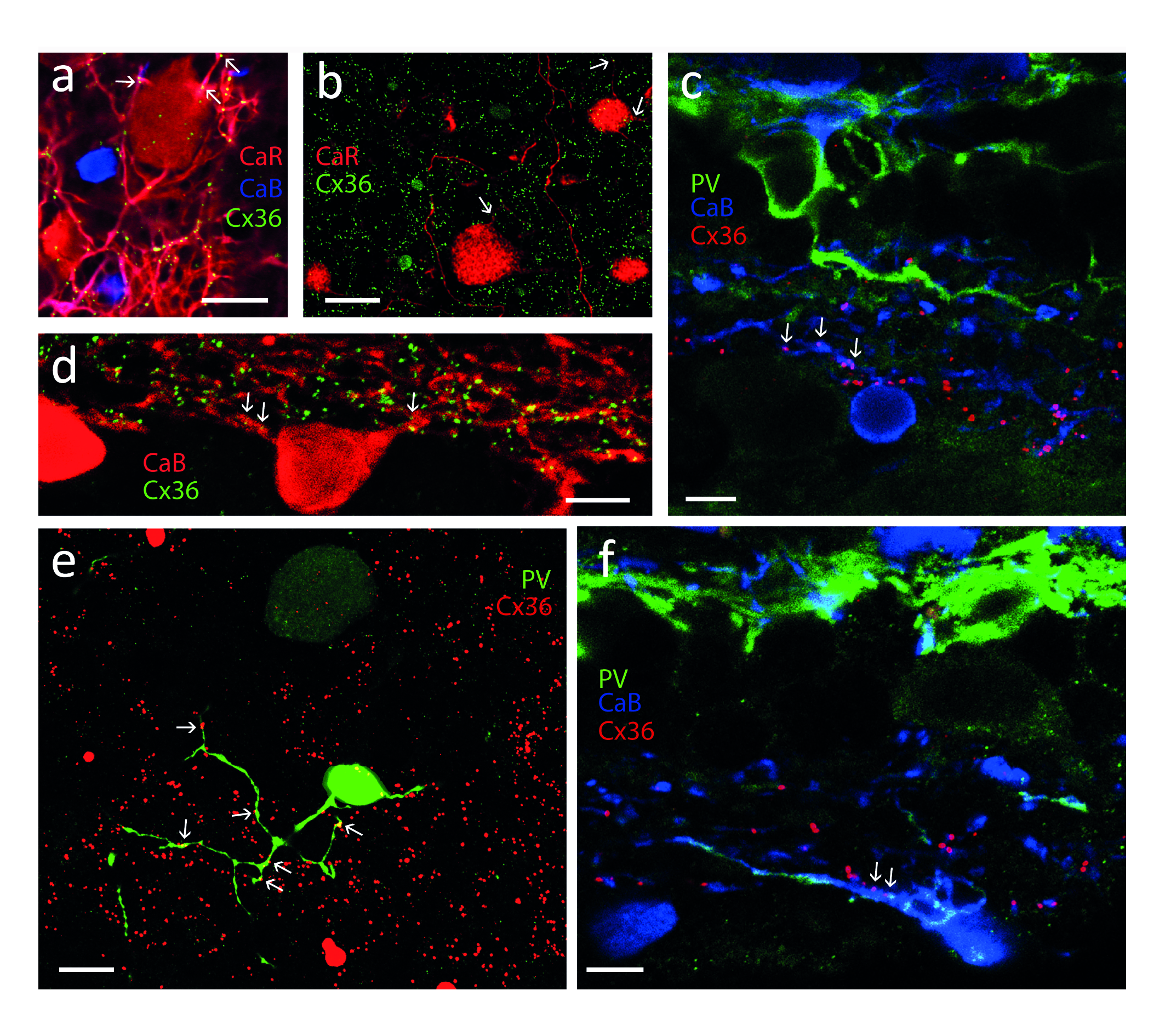

Supplement: Supplemental Figure 4 — (A–F) Images display multiple immunolabels of PV, CaR, CaB, and Cx36. Colocalizing Cx36 plaques can be detected on CaR+ (A,B), on CaB+ (C,D), on PV+ (E) and on CaB/PV dually stained (F) hRGC dendritic processes. It has been shown that PV/CaB dual labeled hRGCs display OFF parasol cell morphology (Kántor et al., 2016b). Scale bars: 20 μm. [file Image_4.tif]
