# Supplementary material for: Strategic Positioning of Connexin36 Gap Junctions Across Human Retinal Ganglion Cell Dendritic Arbors
Source: Front Cell Neurosci. 2018 Nov 22;12:409. doi: 10.3389/fncel.2018.00409 (PMC6262005; doi:10.3389/fncel.2018.00409)
Supplement: Supplemental Table 1 — Table shows measured parameters for all examined hRGCs of this study (left column), the total dendritic lengths (second column, number of all colocalizing Cx36 plaques (third column), the number of Cx36 plaques colocalizing with terminal dendrites, number of all plaque intervals for each cell (fourth column) and the number of plaque intervals < 5 micrometer. [file Table_1.doc]

| **Cells** | **Total Dendrite Length** | **Cx36 Plaque Number** | **Terminal Cx36 Plaque Number** | **Cx36 Plaque Intervals** | **Cx36 Plaque Intervals < 5µm** |
| --- | --- | --- | --- | --- | --- |
| 151109_B_12_1 | 1557,7 | 65 | 26 | 49 | 25 |
| 151109_B_12_2 | 622,7 | 38 | 16 | 23 | 17 |
| 151109_B_12_3 | 631,2 | 73 | 53 | 58 | 35 |
| 151109_B_13_1 | 2110,7 | 60 | 35 | 29 | 20 |
| 151109_B_13_2 | 2219,4 | 66 | 37 | 22 | 12 |
| 151109_B_13_3 | 1766,6 | 84 | 43 | 65 | 30 |
| 151109_B_15_1 | 341,1 | 39 | 29 | 21 | 16 |
| 151109_B_15_2 | 2550,2 | 478 | 289 | 435 | 309 |
| 151109_B_17_1 | 615,9 | 7 | 5 | 0 | 0 |
| 151109_J10_1 | 1671,6 | 141 | 98 | 93 | 56 |
| 151109_J10_2 | 2300,7 | 191 | 162 | 167 | 94 |
| 151109_J12_1 | 1778,6 | 32 | 17 | 15 | 6 |
| 151109_J12_3 | 6898 | 487 | 429 | 448 | 162 |
| 151109_J15_1 | 438 | 9 | 6 | 2 | 1 |
| 151109_J18_1 | 1785,8 | 59 | 37 | 24 | 12 |
| 151109_J18_2 | 1664 | 58 | 41 | 26 | 12 |
| 151109_J18_3 | 2094,2 | 50 | 33 | 24 | 13 |
| 151109_J6_1 | 278 | 6 | 4 | 1 | 0 |
| 151109_J6_2 | 1256,7 | 36 | 19 | 11 | 5 |
| 151109_J6_3 | 760,7 | 24 | 13 | 14 | 8 |
| 151109_J9_1 | 1553,2 | 63 | 45 | 39 | 19 |
| 151109_J9_2 | 1298,8 | 81 | 54 | 50 | 20 |
| 151109_J9_3 | 697,7 | 51 | 40 | 27 | 17 |
| 151123_B13_1 | 2317,3 | 152 | 85 | 80 | 59 |
| 151123_B13_2 | 1726,9 | 107 | 47 | 67 | 49 |
| 151123_B14_1 | 2086,1 | 198 | 135 | 117 | 79 |
| 151123_B14_2 | 1166,8 | 19 | 18 | 10 | 6 |
| 151123_B19_1 | 1097,4 | 98 | 49 | 60 | 41 |
| 151123_B20_1 | 1780,3 | 153 | 77 | 129 | 77 |
| 151123_B20_2 | 1089,3 | 36 | 23 | 10 | 5 |
| 151123_B20_3 | 894,2 | 8 | 7 | 2 | 1 |
| 151123_B20_4 | 621 | 5 | 4 | 3 | 0 |
| 151123_B22_2 | 4832,8 | 177 | 134 | 132 | 36 |
| 151123_B23_1 | 2972,6 | 102 | 57 | 76 | 29 |
| 160107_B5_1 | 2027,7 | 49 | 36 | 19 | 11 |
| 160107_B5_2 | 1851,1 | 66 | 36 | 18 | 12 |
| 160107_B6_1 | 9499,5 | 376 | 197 | 241 | 124 |
| 160107_B6_2 | 2126,6 | 135 | 74 | 65 | 45 |
| 160107_B6_3 | 6650,3 | 438 | 245 | 300 | 138 |
| 160107_B6_4 | 2059,5 | 106 | 79 | 53 | 32 |
| 160107_B6_5 | 1666 | 94 | 41 | 45 | 24 |
| 160107_B6_6 | 1386,3 | 90 | 38 | 73 | 37 |
| 160107_B7_1 | 2575,5 | 184 | 110 | 100 | 73 |
| 160107_B8_1 | 1731,6 | 100 | 53 | 36 | 21 |
| 160107_B8_2 | 2786,2 | 151 | 76 | 75 | 44 |
| 160107_B8_3 | 1923,2 | 54 | 36 | 20 | 9 |
| 160107_B8_4 | 863 | 36 | 19 | 19 | 11 |
